# Atypical Dermatophyte Localization in the Stratum Lucidum: A Retrospective Histopathologic Case Series of Tinea Pedis

**DOI:** 10.3390/jof12100740

**Published:** 2026-10-02

**Authors:** Aditya K. Gupta, Tong Wang, Sara A. Lincoln, Wayne L. Bakotic

**Affiliations:** 1 Division of Dermatology, Department of Medicine, Temerty Faculty of Medicine, University of Toronto, Toronto, ON M5S 3H2, Canada; 2 Division of Dermatology, Women’s College Hospital, Toronto, ON M5S 1B2, Canada; 3 Mediprobe Research Inc., London, ON N5X 2P1, Canada; twang@mediproberesearch.com; 4 Bako Diagnostics, Alpharetta, GA 30005, USA; slincoln@bakodx.com (S.A.L.); wbakotic@bakodx.com (W.L.B.)

**Keywords:** tinea pedis, dermatomycoses, *Trichophyton*, epidermis, stratum lucidum

## Abstract

Dermatophyte infection extending beyond the superficial stratum corneum is an emerging challenge, particularly in immunocompromised patients. *Tinea lucidum*, first described in 2014, reflects this shift in pathogenesis, with dermatophytes localizing in the deeper cornified stratum lucidum and, in some cases, occurring without stratum corneum involvement. This creates diagnostic uncertainty when superficial scrapings are used for mycology testing. We retrospectively analyzed skin punch biopsies submitted to a single U.S. laboratory by dermatologists and podiatrists between January 2024 and March 2026. Specimens were obtained from the plantar and dorsal foot, representing moccasin-type tinea pedis with secondary spread to the dorsal surface. Atypical localization in the stratum lucidum occurred in 43.6% (99/227) of cases, including 13.7% (31/227) without detectable fungal elements in the stratum corneum. The dorsal foot more frequently exhibited tinea lucidum than the plantar foot (76.9% [60/78] vs. 26.2% [39/149]; *p* < 0.0001), potentially reflecting differences in epidermal thickness or underlying immunodeficiency. Higher densities of fungal elements and inflammatory infiltrates revealed by Periodic acid-Schiff and hematoxylin-eosin staining further suggest deeper epidermal invasion; however, the number of samples were limited, and the immune cell populations were not molecularly characterized in this study. For specimens demonstrating dermatophytes localized solely within the stratum lucidum, a survey collected de-identified patient details (responsive rate: 32.3% [10/31]), and sequencing was performed for dermatophyte identification. *Trichophyton rubrum* was identified in the majority of samples tested (64.5% [20/31]), and histories of immunosuppressive medication use and/or immunosuppressive conditions were noted in several patients. Further studies are needed to validate the histologic criteria and elucidate the immunopathogenesis of this phenomenon.

## 1. Introduction

The management of dermatophytosis is facing emerging challenges owing to antifungal resistance and the expanding immunocompromised population [1]. As a common cause of fungal disease affecting up to one-quarter of the world’s population, dermatophyte infections can be categorized based on the affected anatomical site, including tinea capitis, corporis, cruris, unguium (onychomycosis), and pedis, while uncommon clinical presentations are also emerging that reflect a changing epidemiological landscape [2]. Tinea pedis (athlete’s foot) is more commonly diagnosed in older adults, compounded by concomitant hyperhidrosis, obesity, diabetes mellitus, and immunosuppression [1]. It has been generally assumed that the acceleration of urbanization and growth of recreational activities at the turn of the 21st century increased the prevalence of tinea pedis, explained by ill-fitting, occlusive footwear and congregate settings such as public swimming pools and gyms [1].

A summary of clinical variants of tinea pedis is shown in Table 1. Moccasin-type tinea pedis commonly follows a chronic clinical course due in part to the thick stratum corneum of the plantar surface and hyperkeratosis, which may hinder the penetration of topical antifungals and the recruitment of inflammatory infiltrates, reflected by the absence of the eczematous ring-like lesions commonly seen on other types of skin surfaces [3,4]. Additionally, it has been hypothesized that patients develop moccasin-type tinea pedis due to immunodeficiencies or topical corticosteroid use, possibly linked to an atopic background, in which case the infection may disseminate onto the dorsal surface [5,6,7,8]. In these patients, a higher likelihood of observing an immediate-type hypersensitivity response instead of a delayed-type response to *Trichophyton* antigens (trichophytin) reflects skewing toward a non-protective T helper 2 (Th2) response, associated with eosinophil recruitment and elevated IgE levels, which can exacerbate concomitant atopic conditions such as asthma and allergic rhinitis [3,6]. Rarely, dermatophyte invasion of the dermis and subcutaneous tissue may develop in tinea pedis patients following immunosuppression, known as deep dermatophytosis [9].

In immunocompromised patients, a diagnostic challenge—termed *tinea lucidum*—was coined by Mao and colleagues [12]. In their study, difficulty in confirming a dermatophyte etiology by direct microscopic examination led to the discovery of fungal elements localizing within the deeper stratum lucidum layer, and in some cases without the involvement of the stratum corneum. Between 60 and 65% of specimens obtained from non-acral and acral sites showed stratum lucidum involvement; additionally, non-acral sites exhibited more samples in which fungal elements could only be observed in the inferior, deeper aspect of the stratum corneum. In these patients, superficial scraping or sampling of scales may yield false-negative results; therefore, scraping to reveal the inferior stratum corneum or stratum lucidum may be required prior to mycological testing. Some patients had concomitant immunosuppressive conditions, such as ankylosing spondylitis, rheumatoid arthritis, and lupus erythematous, and/or history of taking immunosuppressive medications, such as betamethasone, methylprednisolone, and prednisone.

Herein, we aim to confirm this underrecognized localization of dermatophytosis in the deeper cornified layers and its possible association with immunodeficiencies. In this exploratory study, we conducted retrospective histopathologic examinations of punch biopsy specimens obtained from tinea pedis patients, taken directly from or adjacent to either acral (plantar foot) or non-acral (dorsal foot) sites. For patients whose specimens showed dermatophyte localization solely within the stratum lucidum, a physician survey was distributed to collate additional clinical information, and sequencing was performed for dermatophyte identification.

## 2. Materials and Methods

Between January 2024 and March 2026, records of specimens submitted to a Clinical Laboratory Improvement Amendments (CLIA)-certified molecular diagnostic laboratory (Bako Diagnostics, Alpharetta, GA, USA) were reviewed. The inclusion criteria were 2–3 mm punch biopsy specimens from the lower extremity that were positive for a dermatophyte etiology. Based on the treating dermatologist’s or podiatrist’s request, all specimens were collected in formalin fixative and subsequently subjected to Periodic acid–Schiff (PAS), Grocott’s methenamine silver (GMS), and/or hematoxylin–eosin (H&E) staining. All specimens were submitted as part of a non-interventional, standard-of-care procedure. Secondary, de-identified requisition data—including sex, age, and sampling site—were also reviewed.

All slides (one PAS/GMS and one H&E slide per sample, cut at 4 µm) were examined by a dermatopathologist (W.L.B.). Specimens obtained from the plantar aspect of the foot and the interdigital spaces were classified as acral skin; additionally, specimens obtained near acral surfaces (lateral or medial aspects of the foot) were evaluated individually and designated as acral or non-acral based on the thickness of the stratum corneum and stratum lucidum. Fungal stains were used to assess the localization (i.e., stratum corneum and/or stratum lucidum) and density (i.e., minimal, moderate, or florid) of dermatophytes within the epidermis. For specimens demonstrating dermatophytes localized to the stratum corneum, further empirical evaluations determined whether fungal elements were present within the superior portion, inferior portion, or full thickness of the cornified layer, consistent with the study design of Mao et al. [12]. The stratum lucidum was identified on routine H&E sections by its differential staining pattern, appearing as a translucent layer; in non-acral skin, reactive hyperkeratosis from inflammatory conditions such as tinea infections may produce histologic features consistent with a stratum lucidum. Inflammatory infiltrates identified on H&E staining were categorized by density (i.e., absent, sparse, moderate, or dense), pattern (i.e., superficial perivascular, superficial and deep perivascular, or lichenoid), and character (i.e., acute, chronic, and/or eosinophilic).

Tinea lucidum was defined as the presence of dermatophytes in the stratum lucidum, with or without involvement of the stratum corneum [12]. Data curation and analysis were conducted using Microsoft Excel and IBM SPSS Statistics (version 23). Continuous variables were summarized using the median and range. Categorical variables were summarized using counts and percentages, and statistical comparisons were performed using two-tailed two-proportion Z tests, Fisher’s exact tests, or χ^2^ tests with Bonferroni correction. A *p*-value of ≤0.001 was considered statistically significant.

### 2.1. Physician Survey

A questionnaire was developed and distributed to healthcare providers who submitted specimens demonstrating fungal elements restricted solely within the stratum lucidum. Participants were asked about the patient’s use of immunosuppressive medications and concomitant conditions such as diabetes, onychomycosis, chronic tinea pedis, and chronic pruritus of the feet. They were also asked to describe any unusual skin findings, including lichenification, bullae, pustules, papules, and nodules, as well as the reason for performing a punch biopsy. Lastly, treatment histories—including dosage, duration, and clinical outcome (resolved, improved, no response, or relapsed)—were collected. No patient identifiers were obtained.

### 2.2. ITS and Chitin Synthase Sequencing

Tissue embedded in paraffin blocks was cut into a 10 µm curl, and DNA was extracted using the QIAmp DNA FFPE Advanced Kit—FFPE Tissue DNA extraction (Qiagen [Germantown, MD, USA]; catalog no. 56604) according to the manufacturer’s protocol. The ITS region and the chitin synthase 1 gene were amplified using the Platinum™ PCR SuperMix High Fidelity (Thermo Fisher [Waltham, MA, USA]; catalog no. 12532016) on an ABI7500 thermocycler (Applied Biosystems, Waltham, MA, USA). PCR products were submitted to Genewiz (Azenta Life Sciences, South Plainfield, NJ, USA) for Sanger sequencing.

The ITS region was amplified using primers ITS1F (5′-CTTGGTCATTTAGAGGAAGTAA-3′) and ITS4 (5′-TCCTCCGCTTATTGATATGC-3′) [13,14]. Thermocycling conditions were: 94 °C for 2 min (1 cycle), 94 °C for 30 s, 55 °C for 30 s, and 68 °C for 1 min (40 cycles). For samples with inconclusive ITS sequencing results, the chitin synthase 1 gene was amplified by nested PCR [15]. Primers used for the first round were Chitin synthase_F (5′-CATCGAGTACATGTGCTCGC-3′) and Chitin synthase_R (5′-CTCGAGGTCAAAAGCACGCC-3′). Primers used for the second round were Chitin synthase Nested_F (5′-GCAAAGAAGCCTGGAAGAAG-3′) and Chitin synthase Nested_R (5′-GGAGACCATCTGTGAGAGTTG-3′). Thermocycling conditions for both rounds were: 94 °C for 2 min (1 cycle), 95 °C for 15 s, 60 °C for 30 s, 72 °C for 30 s (5 cycles; autodelta: −1.00) and 94 °C for 15 s, 55 °C for 30 s, 72 °C for 1 min (35 cycles).

Sequence identity was confirmed using BLAST (database: nr; version 2.17.0) with the following thresholds for ITS sequences: E-value ≤ 1 × 10^−100^, percent identity ≥ 90%, and minimum length ≥ 350 bp. For chitin synthase, the thresholds were: E-value ≤ 1 × 10^−60^, percent identity ≥ 90%, and minimum length ≥ 100 bp. A dermatophyte-positive sample required at least one of the two sequencing reactions to meet the above criteria.

## 3. Results

A total of 227 biopsy specimens were retrospectively identified during the study period. This cohort consists of dermatology and podiatry outpatients suspected of tinea pedis and other lower-extremity dermatoses, for whom a 2–3 mm punch biopsy was ordered by the treating physician over the 26-month period. Of these, 65.6% (149/227) were obtained from acral surfaces and 34.4% (78/227) from non-acral surfaces. Acral surfaces were sampled from the plantar aspect of the foot, including forefoot, midfoot, arch, heel, and digits. Non-acral surfaces were sampled from the dorsal aspect of the foot, with an additional 6.4% (5/78) obtained from the ankle and 5.1% (4/78) obtained from the lower leg. The median age was 61 years (range: 14–95), with 41.0% (93/227) males and 59.0% (134/227) females.

### 3.1. Localization of Dermatophytes in the Epidermis and Inflammatory Response

**Figure 1:**
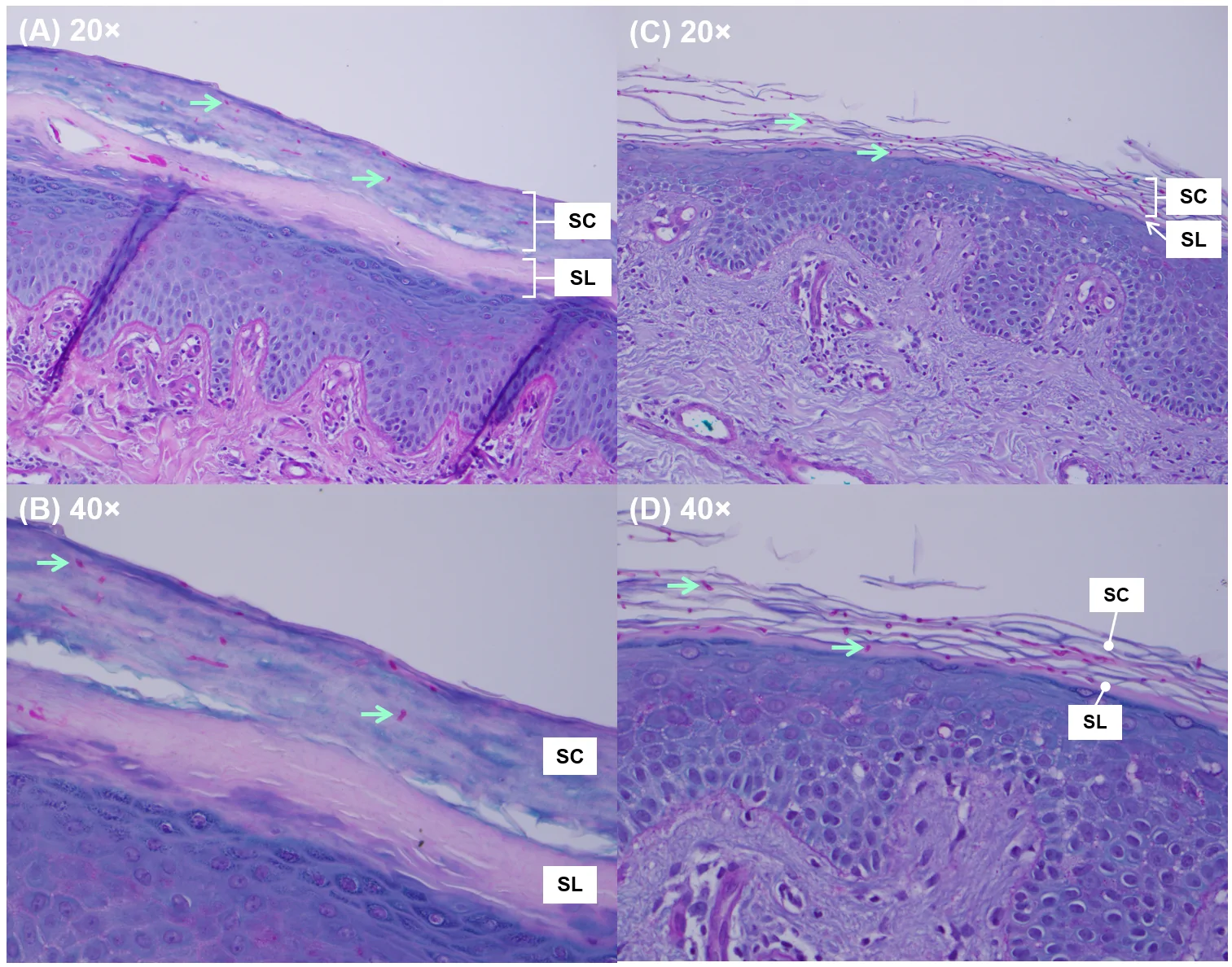
PAS staining showing dermatophytes (**A**,**B**) localized in the stratum corneum from a plantar foot (acral) sample with moderate fungal density, and (**C**,**D**) localized in both the stratum corneum and stratum lucidum (tinea lucidum) from a dorsal foot (non-acral) sample with florid fungal density. Note the differences in the thickness of the cornified layers between acral and non-acral samples. Light green arrows indicate fungal elements. SC, stratum corneum; SL, stratum lucidum.

**Figure 2:**
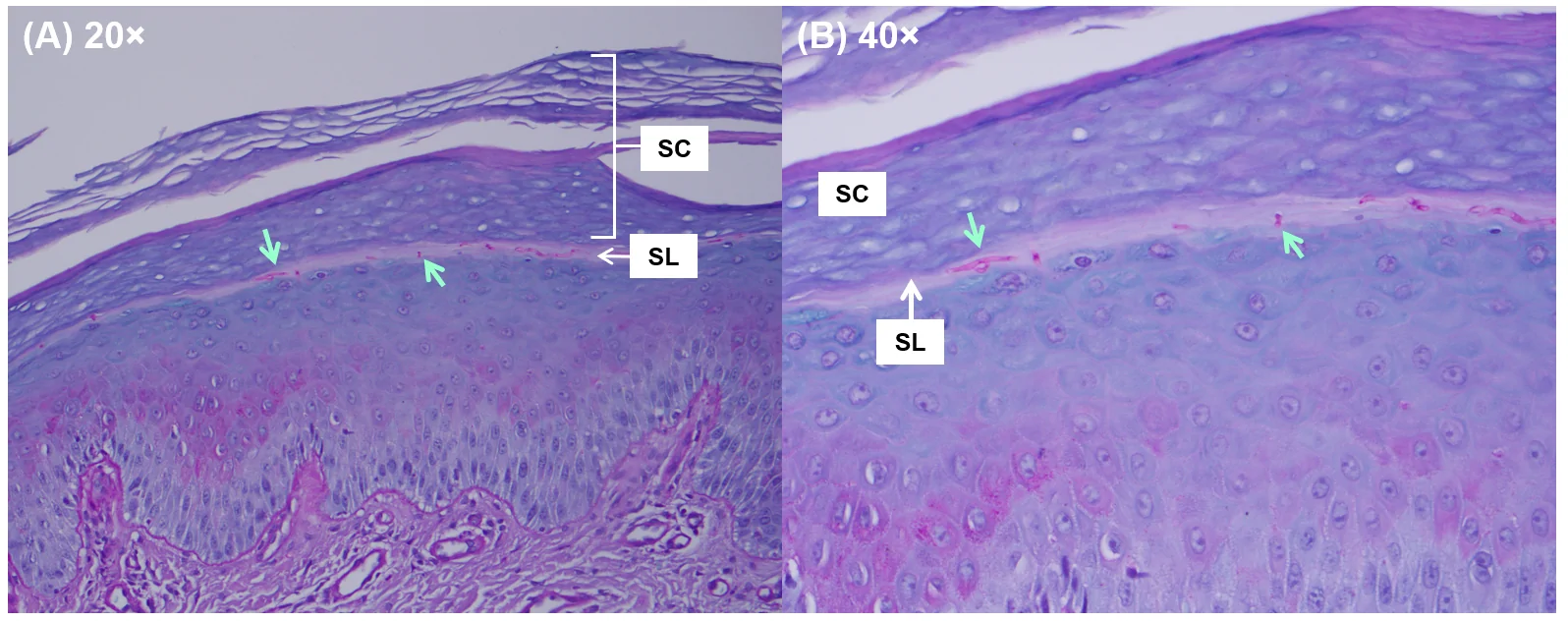
PAS staining showing dermatophytes localized in the stratum lucidum (tinea lucidum) from a dorsal foot (non-acral) sample with moderate fungal density. Note the absence of fungal elements in the stratum corneum. Light green arrows indicate fungal elements. SC, stratum corneum; SL, stratum lucidum.

Atypical dermatophyte localization within the stratum lucidum (tinea lucidum) was identified in 43.6% (99/227) of biopsy specimens obtained from the lower extremity, of which 13.7% (31/227) did not display fungal elements in the stratum corneum (Table 2). Restriction of dermatophytes to the stratum corneum was more frequently observed in acral skin than in non-acral skin (73.8% [110/149] vs. 23.1% [18/78]; *p* < 0.0001). Accordingly, involvement of the stratum lucidum was more commonly observed in non-acral skin than in acral skin (76.9% [60/78] vs. 26.2% [39/149]; *p* < 0.0001). However, these results should be interpreted with caution, as the possibility of selection bias cannot be excluded due to retrospective and convenience sampling. When examining dermatophyte distribution within the stratum corneum, a full thickness pattern was more frequently observed when dermatophyte invasion extended into the stratum lucidum (77.9% [53/68]), compared with cases in which dermatophytes were restricted to the stratum corneum alone (41.4% [53/128]; *p* < 0.0001). Examples of different dermatophyte localization patterns are shown in Figure 1 and Figure 2.

In addition to a higher frequency of stratum lucidum involvement, non-acral skin also exhibited higher fungal densities than acral skin, with 35.9% (28/78) showing florid fungal densities compared with 10.1% (15/149) in acral skin (*p* < 0.0001) (Table 3). Accordingly, minimal fungal densities were more commonly observed in acral skin than in non-acral skin (35.6% [53/149] vs. 20.5% [16/78]; *p* = 0.019). Within non-acral skin, specimens showing dermatophyte localization in the stratum lucidum were also more frequently associated with florid fungal densities (45.9% [17/37] and 34.8% [8/23]) than specimens showing stratum corneum involvement only (16.7% [3/18]), although the sample sizes were limited.

**Figure 3:**
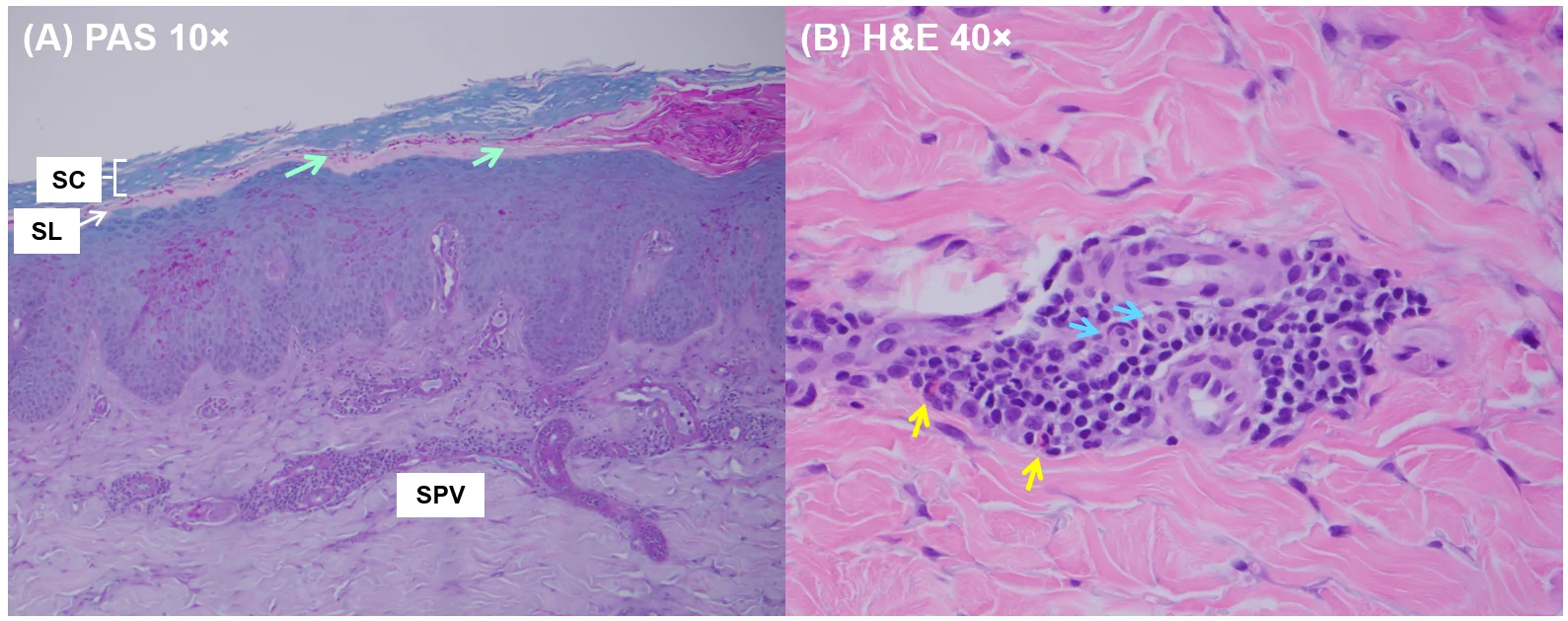
Histopathologic examination of tinea lucidum. (**A**) PAS staining showing florid fungal density along the stratum corneum–stratum lucidum junction and evidence of superficial perivascular inflammation. Light green arrows indicate fungal elements. (**B**) H&E staining showing the presence of eosinophils (yellow arrow) and lymphocytes (blue arrow). SC, stratum corneum; SL, stratum lucidum; SPV, superficial perivascular inflammatory infiltrate.

Corresponding to differences in fungal densities, H&E staining also revealed differences in both the degree and type of inflammatory response observed in acral and non-acral skin. Inflammatory infiltrates were more commonly observed in non-acral skin, with 47.4% (37/78) exhibiting moderate densities compared with 22.8% (34/149) in acral skin (Z = 3.8, *p* < 0.001; Table S1). This pattern became more apparent in non-acral specimens showing dermatophyte invasion of the stratum lucidum; however, the sample size for this subgroup was limited, and no statistical significance was achieved. Across both acral and non-acral skin, inflammation commonly follows a superficial perivascular pattern, whereas superficial and deep perivascular inflammation and lichenoid inflammation were uncommon (Table S2). In 3.8% (3/78) of non-acral specimens, both superficial and deep perivascular inflammation and lichenoid inflammation were present. The composition of inflammatory infiltrates was mostly chronic (e.g., lymphocytes, macrophages) in both acral and non-acral skin (Table S3). However, eosinophilic infiltrates were numerically more common in non-acral skin (11.5% [9/78] vs. 6.0% [9/149]; Z = 1.5, *p* = 0.15), although this difference was not statistically significant. Figure 3A illustrates a case of tinea lucidum with superficial perivascular inflammation and florid fungal densities at the stratum corneum–stratum lucidum junction and Figure 3B reveals the inflammatory composition containing eosinophils and lymphocytes.

### 3.2. Patient Characteristics and Dermatophyte Identification

Of the 31 acral and non-acral specimens in which fungal element was restricted solely to the stratum lucidum, *T. rubrum* was identified in 64.5% (20/31), the *T. mentagrophytes* complex in 6.5% (2/31), and results were inconclusive in 29.0% (9/31), possibly due to low DNA quantities or degradation in FFPE (GenBank: QB045738, QB046943). Clinical information was available for 10 patients, with a survey response rate of 32.2% (10/31; Table 4). Among them, a history of immunosuppressive medication use was found in 5 patients, including topical corticosteroids (*n* = 4), prednisone (*n* = 1), methylprednisolone (*n* = 1), and dupilumab (*n* = 1). Most patients had concomitant chronic tinea pedis and onychomycosis with persistent pruritus. Significant comorbidities that impair host immunity and/or skin barrier integrity include diabetic neuropathy and rheumatoid arthritis.

**Figure 4:**
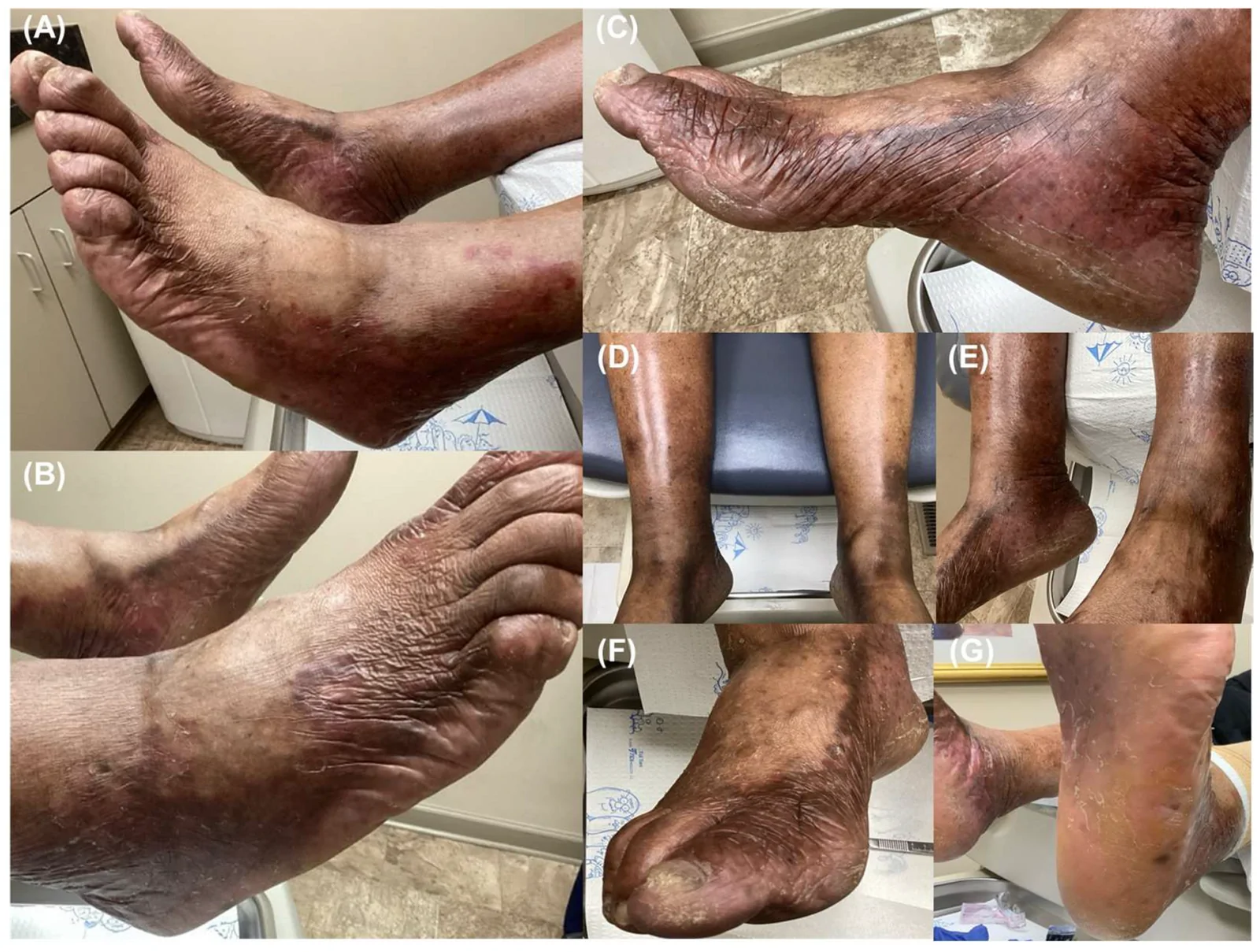
A 79-year-old male patient (patient 4) whose punch-biopsy specimen obtained from the lateral foot (a non-acral site) showed dermatophyte localization (*T. rubrum*) restricted to the stratum lucidum (tinea lucidum). The patient had a history of chronic tinea pedis with persistent pruritus. (**A**–**C**) Xerosis, erythema, and papules are present on the dorsal, medial, and lateral aspects of the feet. (**D**,**E**) The infection progressed to secondary cellulitis. (**F**) Dystrophic toenail changes are present, consistent with onychomycosis, and (**G**) hyperkeratosis of the plantar aspect of the feet is present, consistent with moccasin-type tinea pedis.

**Figure 5:**
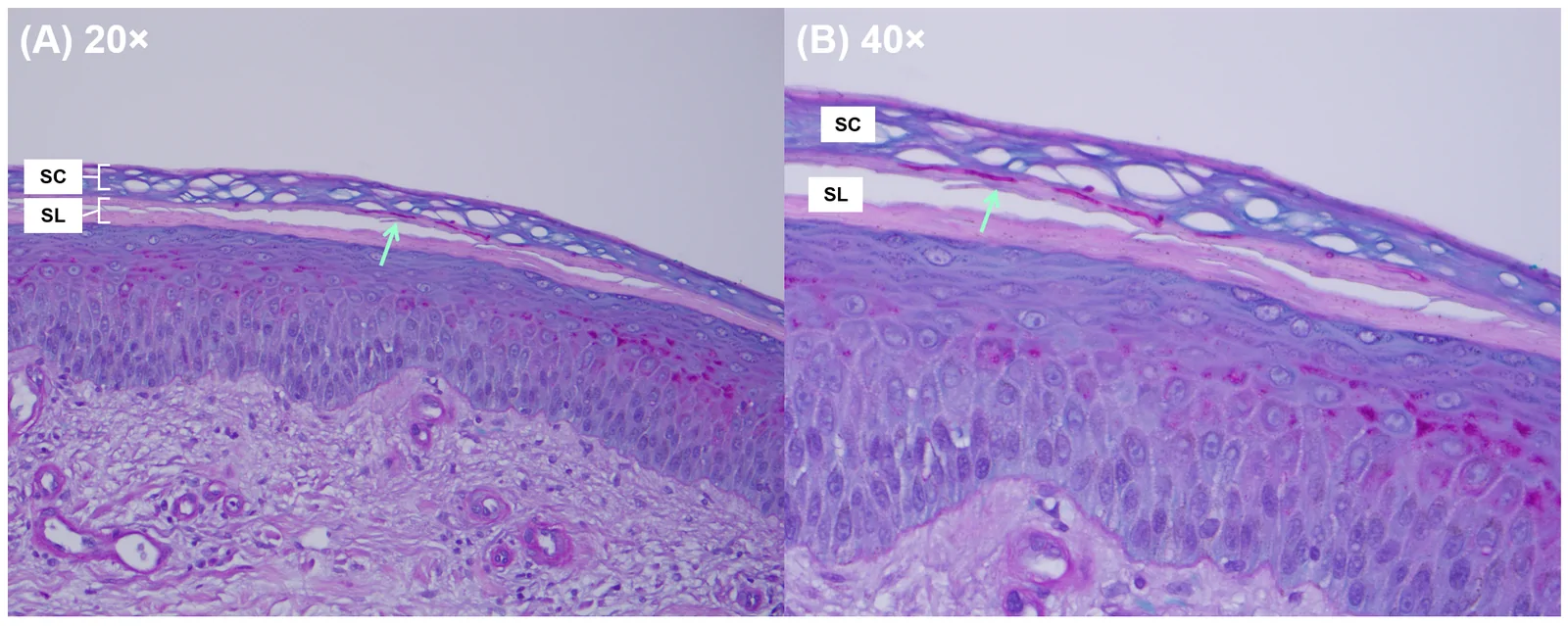
PAS staining of a dorsal foot specimen obtained from patient 4 reveals sparse fungal elements located at the stratum corneum–stratum lucidum junction (tinea lucidum). Light green arrows indicate fungal elements. SC, stratum corneum; SL, stratum lucidum.

Initial treatments mainly consisted of topical antifungals and/or corticosteroids (Table S4). Following histopathologic examination, oral terbinafine (250 mg/d) was tried in 5 patients, with clinical improvement documented in 3; however, the recorded data were incomplete and heterogenous, which precludes systematic evaluation of treatment efficacy. One patient (patient 4; Figure 4) presented with moccasin-type tinea pedis and secondary involvement of the dorsal surface in a bilateral pattern, suggesting an immunocompromised state, although this was not corroborated by survey responses. The dorsal surface exhibited xerosis, marked erythema, and papules; on PAS staining, dermatophytes were identified at the stratum corneum–stratum lucidum junction (Figure 5), which were confirmed as *T. rubrum* by sequencing. After a punch biopsy was performed following the appearance of new lesions, the patient subsequently developed cellulitis of the lower legs. Treatment with terbinafine 250 mg/d and topical clotrimazole 1% resulted in improvement, but relapse occurred.

## 4. Discussion

Tinea pedis is considered the most common clinical variant of dermatophytosis in adults, and chronic infection may develop in immunocompromised patients. Complications can arise from autoinoculation to adjacent skin surfaces (e.g., onychomycosis, tinea cruris), bacterial superinfection causing cellulitis, and tinea manuum (i.e., two-feet-one-hand syndrome) [5,6,9]. In some cases, secondary eruptions on the palm and lateral fingers represent hypersensitivity reactions (i.e., an id reaction, also known as trichophytid or dermatophytid) without the presence of fungal elements, possibly due to hematogenous spread of dermatophyte antigens or activated T lymphocytes originating from the feet [16,17,18]. Additional complications, although rare and not fully defined, include cutaneous (urticaria) and upper airway (bronchial asthma, allergic rhinitis) sensitization through overlapping Th2 pathways, as well as invasive disease such as Majocchi’s granuloma and deep dermatophytosis [5,6,9].

In this study, we highlight another complication in tinea pedis patients, as invasion of the deeper cornified layers was observed in punch biopsies, reflecting a potential shift in dermatophyte pathogenesis. More than half of the specimens examined showed dermatophytes localized within the inferior stratum corneum and/or stratum lucidum, consistent with the observation termed tinea lucidum by Mao and colleagues [12]. In line with their interpretation, tinea lucidum carries a conceivable risk of misdiagnosis at the point of care because standard mycology testing relies on sampling scales or superficial scraping with a blunt edge. If no fungal elements are detected and an empirical diagnosis of atopic dermatitis, psoriasis, or contact dermatitis is made, subsequent topical steroid use may alter lesion morphology, further complicating clinical diagnosis, and induce local immunosuppression that potentially enables dermatophytes to move deeper into the epidermis. Accordingly, the fact that punch biopsies were ordered for these patients suggests diagnostic uncertainty, possibly compounded by a history of topical steroid use or other immunosuppressive medications for managing comorbidities. Due to the lack of complete clinical information and treatment histories, we caution that this observation alone does not suggest a change in treatment indication from topical to systemic antifungals, but rather highlights a potential diagnostic challenge.

Additionally, despite efforts to include all specimens submitted for histopathologic examination to a CLIA-certified laboratory, the specimens identified in this study were taken from the plantar or dorsal foot, while interdigital (web space) specimens were notably absent. This observation may be explained by differences in sampling-site accessibility in cases of mixed-type tinea pedis, as well as by the chronic nature of moccasin-type tinea pedis, which, when exacerbated by local or systemic immunodeficiencies, is thought to spread to the dorsal aspect of the foot [3,5]. When comparing the dorsal foot (non-acral) to the plantar foot (acral), tinea lucidum was observed in a significantly higher proportion of specimens, possibly due to a difference in epidermal thickness. A higher density of fungal elements may also reflect a dysregulated immune response. Consistent with this interpretation, specimens obtained from the dorsal foot demonstrated a greater density of inflammatory infiltrates. Although the analysis precluded complete patient information and systematic immunological characterization, a history of immunosuppressive medication use or immunosuppressive conditions was evident in some of the tinea lucidum patients. Further studies are warranted to examine the underlying role of immunodeficiencies.

In the literature, moccasin-type tinea pedis has been associated with subclinical and clinical immunocompromised states. Trichophytin patch testing has shown a significantly lower positivity rate among patients with moccasin-type tinea pedis compared with those with interdigital or vesiculobullous tinea pedis, reflecting dysregulation of cell-mediated immunity [4]. Accordingly, positivity rates were similarly reduced in patients with tinea corporis complicated by steroid use and in cases of tinea incognito [4]. Comparable findings have been reported with intradermal trichophytin testing, in which patients with moccasin-type tinea pedis—characterized by chronic, persistent infection—more frequently exhibited an immediate-type hypersensitivity response, reflective of humoral immunity and a skewed Th2-type profile, with increased susceptibility to reinfection compared with those with a history of acute resolution [19,20].

Although dermatophytes are considered obligate keratinophilic fungi that restrict their growth solely to the non-viable epidermis [21], an ultrastructural analysis of tinea pedis identified degenerative changes throughout the epidermis compared with healthy controls [22]. These findings included disruption of intercellular junctions and widened spacing between keratinocytes and between basal cells, potentially facilitating the penetration of polymorphonuclear leukocytes (neutrophils) and lymphocytes (Th1, Th17 cells). Nuclear and cytoskeletal alterations in keratinocytes were also observed, along with round granules resembling lamellar bodies, which may indicate the release of antimicrobial peptides. Accordingly, an immunohistochemical study found human β-defensins 2 and 3 to be highly expressed in the stratum corneum of dermatophytosis patients compared with the deeper stratum spinosum and stratum basale layers [23]. Increased epidermal proliferation, reflected by a shortened transit time for basal cells to migrate upward through the stratum granulosum and into the stratum corneum before desquamation, is also evident as an innate immune response and manifests clinically as scaling [24,25]. Furthermore, in the absence of immunity, *T. rubrum* infection of skin explants has been shown to invade the full-thickness epidermis and dermis [26,27], possibly through the production of proteases targeting keratin and elastin [28]. Together, these observations suggest that host immunity plays a role in confining dermatophyte infections to the stratum corneum, and that dysregulation of this response may permit infection to extend into the deeper epidermis.

### Study Limitations

The results presented in this study are limited by retrospective data collection, convenience sampling from a single laboratory, histopathologic examinations performed by a single observer without blinding, and lack of clinical characterization and treatment details. Although we highlight tinea lucidum as an emerging diagnostic challenge using a large collection of punch biopsy specimens, its histologic criteria have not been established with a scientific consensus. Additionally, we could not definitely exclude the possibility of sampling artifacts when fungal elements are observed in the inferior stratum corneum and/or stratum lucidum. The patient cohort included here consists of dermatology and podiatry outpatients with tinea pedis who underwent a 2–3 mm skin punch biopsy performed by the treating physician, which likely overrepresents patients with greater disease severity and comorbidity burden compared with the general outpatient population. Although we note that some of the tinea lucidum patients were possibly immunocompromised, immunosuppression status was not systematically defined or measured in this study. This interpretation is also limited by the lack of a control group (e.g., interdigital tinea pedis) and the small sample size available for subgroup analyses.

Potential future studies include ultrastructural analyses of tissues obtained from tinea lucidum patients and control patients, along with clinical and laboratory evaluations for primary or acquired immunodeficiencies. These may include molecular characterization of infiltrating immune cell populations (Th1/Th17, Th2) and alterations in signaling pathways (e.g., CARD9 deficiency) [29]. Alternatively, a reproducible animal infection model can also help to elucidate the immunopathogenesis of tinea lucidum [30].

## 5. Conclusions

Dermatophytosis is canonically defined as a superficial infection of the stratum corneum affecting the skin, hair, and nails [1]. Ever since the global spread of *T. rubrum*, the literature has documented clinical observations of some patients becoming chronically infected, and, in rare instances, developing deep, disseminated infections associated with primary and acquired immunodeficiencies [9,31]. With the expansion of immunotherapies that have revolutionized medicine and shifted host characteristics, the pathogenesis of dermatophytes—obligate human pathogens that are unique within the fungal kingdom and representing one of the oldest infectious diseases known to humans—warrants re-examination [32]. In this work, we highlight dermatophyte invasion of the stratum lucidum, a deeper cornified layer, in patients presenting with moccasin-type tinea pedis and in patients where the dorsal foot is also affected. This observation, termed tinea lucidum, can be challenging to diagnose, as the fungal element may reside within the inferior stratum corneum or exclusively in the stratum lucidum. Further studies are warranted to examine the role of immunodeficiencies in dermatophyte pathogenesis.

## Figures and Tables

**Table 1:** Clinical variants of tinea pedis.

Clinical Variant	Description
Interdigital tinea pedis	The most common manifestation of tinea pedis, whereby maceration of the webspace occurs, with a risk of secondary bacterial colonization—such as *Corynebacterium* and *Pseudomonas*—due to the warm, humid microenvironment supporting a polymicrobial flora [3,10,11].
Ulcerative tinea pedis	In interdigital tinea pedis, bacterial colonization may cause further maceration and fissuring, which can subsequently progress to ulceration.
Moccasin-type tinea pedis	The second most common clinical variant, in which hyperkeratosis is observed at the plantar aspect. It ranges from minimal scaling to diffuse hyperkeratosis and may extend onto the lateral and medial surfaces in a bilateral pattern [5,6]. Fissures may develop within hyperkeratotic plaques and can impair ambulation.
Vesiculobullous tinea pedis	Primarily associated with the *Trichophyton mentagrophytes* complex and is sometimes also referred to as dyshidrotic tinea pedis. It is characterized by acute inflammatory eruptions with intense pruritus, burning, and pain [5,6]. Secondary bacterial superinfection may occur when pustules are apparent.

**Table 2:** Dermatophyte distribution within the epidermis in acral and non-acral skin.

Epidermal Localization, % (*n*)	Total (*n* = 227)	Acral Sites (*n* = 149)	Non-Acral Sites (*n* = 78)	*p*-Value *
Stratum Corneum	56.4 (128)	73.8 (110)	23.1 (18)	<0.0001 ^†^
Stratum Corneum Localization				
Superior	40.6 (52)	40.0 (44)	44.4 (8)	NS
Inferior	18.0 (23)	17.3 (19)	22.2 (4)	
Full Thickness	41.4 (53)	42.7 (47)	33.3 (6)	
Stratum Corneum and Stratum Lucidum	30.0 (68)	20.8 (31)	47.4 (37)	<0.0001 ^‡^
Stratum Corneum Localization				
Superior	0	0	0	NS
Inferior	22.1 (15)	19.4 (6)	24.3 (9)	
Full Thickness	77.9 (53)	80.6 (25)	75.7 (28)	
Stratum Lucidum	13.7 (31)	5.4 (8)	29.5 (23)	<0.0001 ^§^

NS, not significant. * Statistical comparisons between acral and non-acral sites were performed using two-tailed, two-proportion Z tests, Fisher’s exact tests or χ^2^ tests with Bonferroni correction. ^†^ Z test statistic: 7.3; *p* = 2.5 × 10^−13^. ^‡^ Z test statistic: −4.2; *p* = 3.2 × 10^−5^. ^§^ Z test statistic: −5.0; *p* = 5.0 × 10^−7^.

**Table 3:** Comparison of fungal densities between acral and non-acral skin, stratified by epidermal localization patterns.

Epidermal Fungal Localization	Fungal Density, % (*n*)			*p*-Value *
	Minimal	Moderate	Florid	
Acral Sites				
Total (*N* = 149)	35.6 (53)	54.4 (81)	10.1 (15)	<0.0001 ^†^
Stratum Corneum (*N* = 110)	40.0 (44)	52.7 (58)	7.3 (8)	NS
Stratum Corneum and Stratum Lucidum (*N* = 31)	19.4 (6)	61.3 (19)	19.4 (6)	
Stratum Lucidum (*N* = 8)	37.5 (3)	50.0 (4)	12.5 (1)	
Non-Acral Sites				
Total (*N* = 78)	20.5 (16)	43.6 (34)	35.9 (28)	<0.0001 ^‡^
Stratum Corneum (*N* = 18)	61.1 (11)	22.2 (4)	16.7 (3)	<0.0001 ^§^
Stratum Corneum and Stratum Lucidum (*N* = 37)	2.7 (1)	51.4 (19)	45.9 (17)	
Stratum Lucidum (*N* = 23)	17.4 (4)	47.8 (11)	34.8 (8)	

NS, not significant. * Statistical comparisons between fungal density categories—both within acral and non-acral sites or further stratified by the epidermal fungal localization patterns—were performed using two-tailed Fisher’s exact tests or χ^2^ tests with Bonferroni correction. ^†^ χ^2^ test statistic: 44.2, *p* = 2.5 × 10^−10^. ^‡^ χ^2^ test statistic: 37.2, *p* = 8.3 × 10^−9^. ^§^ Fisher’s exact test statistic: 23.1; *p* = 7.2 × 10^−5^.

**Table 4:** Characteristics of tinea pedis patients detected with fungal elements restricted solely within the stratum lucidum (tinea lucidum).

Pt No.	Age/Sex	Immunosuppressive Medication	Concomitant Condition	Skin Finding and Reason for Biopsy	Dermatophyte
1	52/M	No	Onychomycosis Chronic tinea pedis	Xerosis and erythema; R/O dermatitis	ND
2	73/F	No	Diabetes Onychomycosis Chronic tinea pedis and pruritus	R/O tinea pedis	ND
3	83/M	NR	NR	Scaling on the plantar foot with erythematous macules	*T. rubrum*
4	79/M	No	Onychomycosis Chronic tinea pedis and pruritus Cellulitis S/P punch biopsy	New lesion	*T. rubrum*
5	74/M	Yes (Topical steroids)	Diabetes with neuropathy Chronic tinea pedis and pruritus	Xerosis, pink/red papules on dorsal foot in circinate and serpiginous patterns, Schamberg’s presentation on legs.	*T. rubrum*
6	83/F	No	Onychomycosis Chronic tinea pedis and pruritus	Xerosis, hyperkeratosis, scaling; R/O eczema, tineas	*T. rubrum*
7	75/F	Yes (Topical steroids) (Methylprednisolone)	Diabetes Rheumatoid arthritis Onychomycosis Chronic tinea pedis and pruritus	Chronic epidermal skin peeling and rash	ND
8	58/M	Yes (Topical steroids) (Prednisone)	Chronic headaches Onychomycosis Chronic tinea pedis and pruritus	Chronic skin changes not responsive to topical and oral treatments	*T. rubrum*
9	69/F	Yes (Dupilumab)	Hypertension Adrenoleukodystrophy Osteoarthritis Asthma Onychomycosis Chronic tinea pedis	Failed topical antifungals and steroids	ND
10	78/F	Yes (Topical steroids)	Onychomycosis Chronic tinea pedis and pruritus	Chronic dorsal foot rash not responding to topical antifungals or steroids	*T. rubrum*

ND, not determined; NR, not reported; R/O, rule out; S/P status post.

## Data Availability

Restrictions apply to the availability of these data. Data were obtained from Bako Diagnostics (Alpharetta, GA, USA) and are available from author A.K.G. with the permission of Bako Diagnostics.

## Supplementary Materials

The following supporting information can be downloaded at https://www.mdpi.com/article/10.3390/jof12100740/s1. Table S1: Comparison of inflammatory infiltrate densities between acral and non-acral skin, stratified by epidermal localization patterns; Table S2: Comparison of inflammatory infiltrate patterns between acral and non-acral skin, stratified by epidermal localization patterns; Table S3: Comparison of inflammatory infiltrate compositions between acral and non-acral skin, stratified by epidermal localization patterns; Table S4: Treatment histories of tinea pedis patients detected with fungal elements restricted solely within the stratum lucidum (tinea lucidum).
