# Supplementary material for: Atypical Dermatophyte Localization in the Stratum Lucidum: A Retrospective Histopathologic Case Series of Tinea Pedis
Source: J Fungi (Basel). 2026 Oct 2;12(10):740. doi: 10.3390/jof12100740 (PMC13706480; doi:10.3390/jof12100740)
Supplement: Supplementary file 1 [file jof-12-00740-s001.zip › jof-4557260-supplementary.pdf]

Atypical Dermatophyte Localization in the Stratum Lucidum: A Retrospective Histopathologic Case Series of Tinea Pedis

Aditya K. Gupta, Tong Wang, Sara A. Lincoln, and Wayne L. Bakotic

Journal of Fungi

Supplementary Materials

Table of Contents

Table S1. Comparison of inflammatory infiltrate densities between acral and non-acral skin, stratified by epidermal localization patterns. 2

Table S2. Comparison of inflammatory infiltrate patterns between acral and non-acral skin, stratified by epidermal localization patterns. 3

Table S3. Comparison of inflammatory infiltrate compositions between acral and non-acral skin, stratified by epidermal localization patterns.. 4

Table S4. Treatment histories of tinea pedis patients detected with fungal elements restricted solely within the stratum lucidum (tinea lucidum).. 5

Table S1. Comparison of inflammatory infiltrate densities between acral and non-acral skin, stratified by epidermal localization patterns.

**Epidermal Fungal Localization**	**Density of Inflammatory Infiltrates, % (n)**				***p*-value***
	**Absent**	**Sparse**	**Moderate**	**Dense**	
Acral Sites					
Total (N=149)	21.5 (32)	46.3 (69)	22.8 (34)	6.7 (10)	<0.0001†
Stratum Corneum (N=110)	22.7 (25)	48.2 (53)	20.9 (23)	4.5 (5)	NS
Stratum Corneum and Stratum Lucidum (N=31)	22.6 (7)	41.9 (13)	25.8 (8)	9.7 (3)	
Stratum Lucidum (N=8)	0	37.5 (3)	37.5 (3)	25.0 (2)	
Non-Acral Sites					
Total (N=78)	5.1 (4)	46.2 (36)	47.4 (37)	1.3 (1)	<0.0001‡
Stratum Corneum (N=18)	11.1 (2)	50.0 (9)	38.9 (7)	0	NS
Stratum Corneum and Stratum Lucidum (N=37)	5.4 (2)	40.5 (15)	51.4 (19)	2.7 (1)	
Stratum Lucidum (N=23)	0	52.2 (12)	47.8 (11)	0	

Four acral specimens were excluded due to indeterminate results.

NS, not significant.

*Statistical comparisons between fungal density categories were performed using two-tailed Fisher's exact tests or χ^2 tests with Bonferroni correction.

†χ^2 statistic: 164.6; $p = 1.9\times10^{-35}$

‡χ^2 statistic: 63.0; $p = 1.4\times10^{-13}$

Table S2. Comparison of inflammatory infiltrate patterns between acral and non-acral skin, stratified by epidermal localization patterns.

Epidermal Fungal Localization	Pattern of Inflammatory Infiltrates, % (n)				*p*-value*
	SPV	SPV/L	SDPV	SDPV/L	
Acral Sites					
Total (N=149)	64.4 (96)	1.3 (2)	10.1 (15)	0	<0.0001†
Stratum Corneum (N=110)	65.5 (72)	0.9 (1)	7.3 (8)	0	NS
Stratum Corneum and Stratum Lucidum (N=31)	61.3 (19)	3.2 (1)	12.9 (4)	0	
Stratum Lucidum (N=8)	62.5 (5)	0	37.5 (3)	0	
Non-Acral Sites					
Total (N=78)	73.1 (57)	1.3 (1)	16.7 (13)	3.8 (3)	<0.0001‡
Stratum Corneum (N=18)	77.8 (14)	0	11.1 (2)	0	NS
Stratum Corneum and Stratum Lucidum (N=37)	64.9 (24)	0	27.0 (10)	2.7 (1)	
Stratum Lucidum (N=23)	82.6 (19)	4.3 (1)	4.3 (1)	8.7 (2)	

Forty specimens are not shown due to the lack of inflammatory response.

L, lichenoid; NS, not significant; SPV, superficial perivascular; SDPV, superficial and deep perivascular

*Statistical comparisons between fungal density categories were performed using two-tailed Fisher's exact tests or χ^2 tests with Bonferroni correction.

†χ^2 statistic: 358.5; $p = 2.1\times10^{-77}$

‡χ^2 statistic: 111.3; $p = 5.8\times10^{-24}$

Table S3. Comparison of inflammatory infiltrate compositions between acral and non-acral skin, stratified by epidermal localization patterns.

Epidermal Fungal Localization	Character of Inflammatory Infiltrates, % (n)				*p*-value*
	**A/C**	**A/C/E**	**C**	**C/E**	
Acral Sites					
Total (N=149)	3.4 (5)	1.3 (2)	65.1 (97)	6.0 (9)	<0.0001†
Stratum Corneum (N=110)	2.7 (3)	1.8 (2)	64.5 (71)	4.5 (5)	NS
Stratum Corneum and Stratum Lucidum (N=31)	3.2 (1)	0	67.7 (21)	6.5 (2)	
Stratum Lucidum (N=8)	12.5 (1)	0	62.5 (5)	25.0 (2)	
Non-Acral Sites					
Total (N=78)	6.4 (5)	1.3 (1)	75.6 (59)	11.5 (9)	<0.0001‡
Stratum Corneum (N=18)	5.6 (1)	0	72.2 (13)	11.1 (2)	NS
Stratum Corneum and Stratum Lucidum (N=37)	5.4 (2)	2.7 (1)	73.0 (27)	13.5 (5)	
Stratum Lucidum (N=23)	8.7 (2)	0	82.6 (19)	8.7 (2)	

Forty specimens are not shown due to the lack of inflammatory response.

A, acute; C, chronic; E, eosinophil; NS, not significant.

*Statistical comparisons between fungal density categories were performed using two-tailed Fisher's exact tests or χ^2 tests with Bonferroni correction.

†χ^2 statistic: 362.5; $p = 2.9\times10^{-78}$

‡χ^2 statistic: 119.9; $p = 7.9\times10^{-26}$

Table S4. Treatment histories of tinea pedis patients detected with fungal elements restricted solely within the stratum lucidum (tinea lucidum).

Pt	Before/After Biopsy	Treatment (Chronological Order)	Regimen	Treatment Response
1	After	Topical ciclopirox	Twice daily for 6 weeks	Relapsed
	After	Topical ciclopirox	Twice daily for 6 weeks	Resolved
2	After	Topical ketoconazole 2%	2 weeks	Resolved
3	After	Terbinafine	NR	Improved
	After	Topical ketoconazole 2%	NR	NR
4	Before	Topical tolnaftate 1% + urea 40%	3 weeks	No response
	After	Oral Terbinafine	250 mg/d for 2 months	Improved
	After	Topical clotrimazole 1%	2 months	Improved then relapsed
	After	Oral Terbinafine	250 mg/d for 1 month	Improved then relapsed
5	Before	Fluocinonide cream 0.05%	Two to three times daily for 2 weeks	No response
	After	Clotrimazole cream	Twice daily for 6 weeks	Resolved
6	NR	NR	NR	NR
7	Before	Methylprednisolone dose pack	1 week	Improved
	Before and After	Topical triamcinolone 0.5%	Twice daily	No response
	Before and After	Oral terbinafine	250 mg/d for 6 weeks	Improved
8	Before	Terbinafine	NR	No response
	Before	Topical clobetasol	NR	No response
	Before	Oral prednisone for headaches	NR	NR
	After	Topical tolnaftate 1% for skin	1 month	Resolved
	After	Topical antifungal + urea for nail	1 year	NR
9	Before	Topical oxiconazole	1 month	No response
	After	Oral terbinafine + Topical oxiconazole	NR	Improved
10	Before	Topical antifungals and steroids	NR	Improved then relapsed
	After	Topical terbinafine	Twice daily for 4-6 weeks	NR

NR, not reported
